# (*E*)-2-(Isonicotinoylhydrazonometh­yl)benzoic acid methanol monosolvate

**DOI:** 10.1107/S1600536809038902

**Published:** 2009-09-30

**Authors:** Wenkuan Li, Handong Yin, Liyuan Wen, Jichun Cui, Daqi Wang

**Affiliations:** a College of Chemistry and Chemical Engineering, Liaocheng University, Shandong 252059, People’s Republic of China

## Abstract

The title compound, C_14_H_11_N_3_O_3_·CH_4_O, was synthesized by the condensation reaction of isonicotinohydrazide with an equimolar quantity of 2-formyl­benzoic acid in methanol. The hydrazone mol­ecule displays an *E* configuration about the C=N bond. The dihedral angel between the pyridine and the benzene rings is 12.04 (5)°. In the crystal structure, mol­ecules are linked by O—H⋯N, O—H⋯O and N—H⋯O hydrogen-bonding inter­actions.

## Related literature

For general background to hydrazones, see: Dhande *et al.* (2007 ▶). For a related structure, see: Zhang *et al.* (2009 ▶).
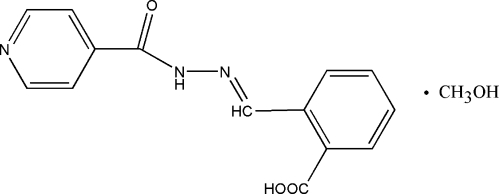


## Experimental

### 

#### Crystal data

C_14_H_11_N_3_O_3_·CH_4_O
*M* *_r_* = 301.30
Monoclinic, 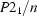

*a* = 6.9768 (11) Å
*b* = 12.2103 (13) Å
*c* = 17.2650 (19) Å
β = 95.497 (1)°
*V* = 1464.0 (3) Å^3^
*Z* = 4
Mo *K*α radiation
μ = 0.10 mm^−1^
*T* = 298 K
0.43 × 0.19 × 0.15 mm

#### Data collection

Siemens SMART CCD area-detector diffractometer
Absorption correction: multi-scan (*SADABS*; Sheldrick, 1996 ▶) *T*_min_ = 0.958, *T*_max_ = 0.985
7290 measured reflections
2508 independent reflections
1233 reflections with *I* > 2σ(*I*)
*R*_int_ = 0.076

#### Refinement

*R*[*F* ^2^ > 2σ(*F* ^2^)] = 0.050
*wR*(*F* ^2^) = 0.141
*S* = 0.99
2508 reflections
199 parameters
H-atom parameters constrained
Δρ_max_ = 0.34 e Å^−3^
Δρ_min_ = −0.19 e Å^−3^

### 

Data collection: *SMART* (Siemens, 1996 ▶); cell refinement: *SAINT* (Siemens, 1996 ▶); data reduction: *SAINT*; program(s) used to solve structure: *SHELXS97* (Sheldrick, 2008 ▶); program(s) used to refine structure: *SHELXL97* (Sheldrick, 2008 ▶); molecular graphics: *SHELXTL* (Sheldrick, 2008 ▶); software used to prepare material for publication: *SHELXTL*.

## Supplementary Material

Crystal structure: contains datablocks I, global. DOI: 10.1107/S1600536809038902/bq2160sup1.cif

Structure factors: contains datablocks I. DOI: 10.1107/S1600536809038902/bq2160Isup2.hkl

Additional supplementary materials:  crystallographic information; 3D view; checkCIF report

## Figures and Tables

**Table 1:** Hydrogen-bond geometry (Å, °)

*D*—H⋯*A*	*D*—H	H⋯*A*	*D*⋯*A*	*D*—H⋯*A*
N1—H1⋯O4^i^	0.86	2.13	2.891 (3)	148
O4—H4⋯O1	0.82	2.14	2.864 (4)	148
O2—H2⋯N3^ii^	0.82	1.76	2.565 (3)	165

Symmetry codes: (i) 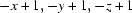; (ii) 
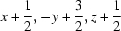
.

**Fig. 1.**
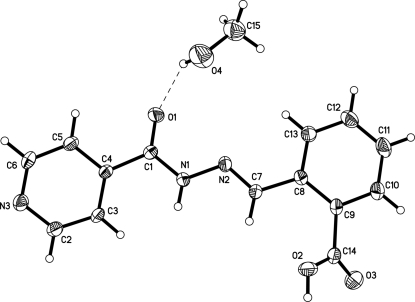
The molecular structure (I) with 50% probability displacement ellipsoids. O—H···O hydrogen bond is shown in dashed line.

**Fig. 2.**
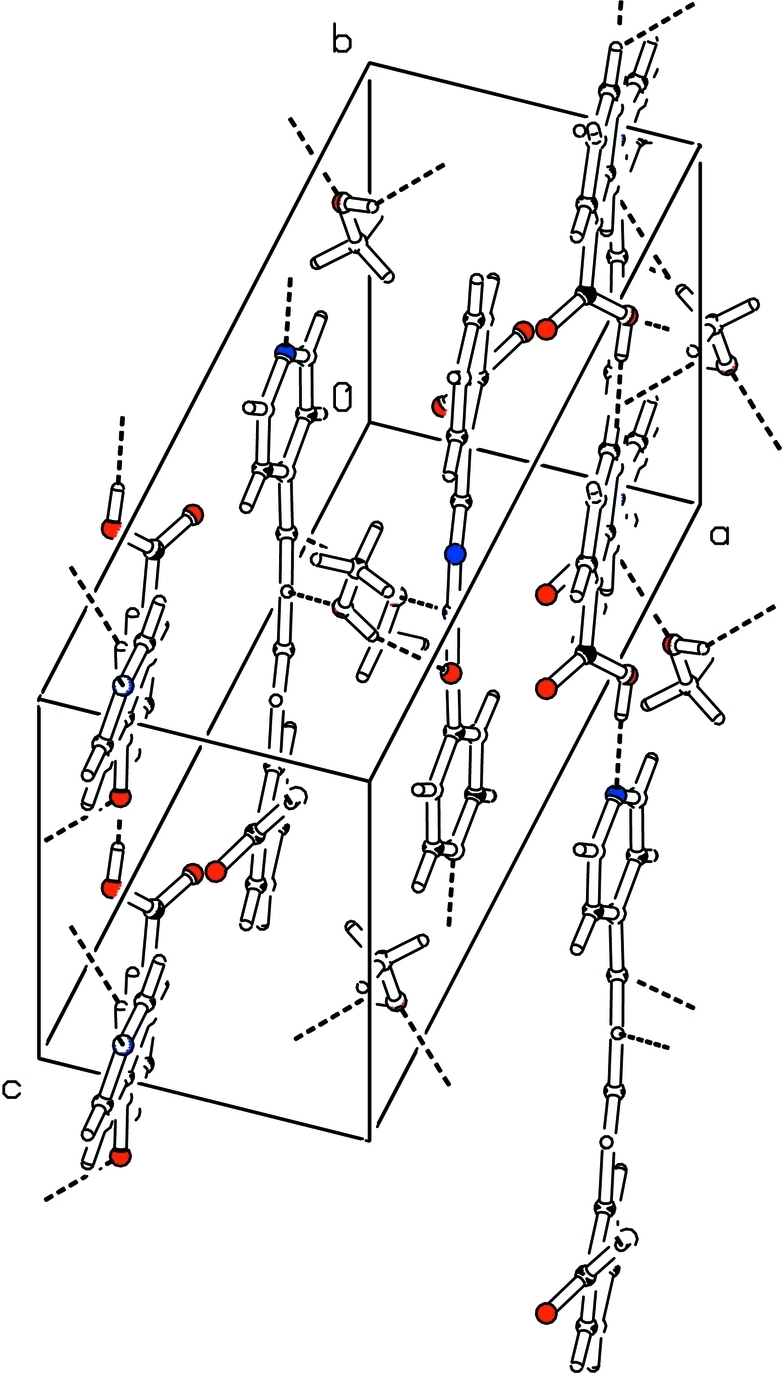
The molecular packing of the title compound. Hydrogen bonding is shown in dashed lines.

## supplementary crystallographic information

### Comment

Hydrazones have been attracted significant attention because of their physiological activity, coordinative capability, and applications in analytical chemistry (Dhande *et al.* 2007). Recently, a large number of hydrazone compounds have been reported (Zhang *et al.* 2009). As a contribution to the chemistry of hydrazone, we report here the synthesis and crystal structure of the title compound (I).

The crystal structure of (I) is built up of hydrazone and methanol molecules (Fig.1). The dihedral angel between the pyridine and the benzene rings is 12.04 (5) °. The hydrazone molecule crystallizes in E conformation. In the crystal structure, three kinds of intermolecular O—H···N, O—H···O and N—H···O hydrogen bonding interactions are observed and the crystal packing is stabilized by these intermolecular interactions. (Table 1. and Fig. 2).

### Experimental

Isonicotinohydrazide (10 mmol) was dissolved in ethanol (40 ml), then 2-formylbenzoic acid (10 mmol) was added into the solution. The reaction mixture was heated under reflux for 2 h. After the solution had cooled to room white sediment appeared. The product was crystallized from methanol. Anal. Calcd (%) for [(C_14_H_11_N_3_O_3_).(C_1_H_4_O_1_)] (Mr = 301.30): C, 59.79; H, 5.02; N, 13.95; O, 21.24 Found (%): C, 59.83; H, 5.00; N, 13.92; O, 21.25

### Refinement

The imino H atom was located in a difference Fourier map and refined isotropically, with the N—H distance restrained to 0.86 Å. Other H atoms were positioned geometrically and constrained to ride on their parent atoms, with C—H = 0.93 (aromatic and methylene) and 0.96(methyl), O—H = 0.82, and with *U*_iso_(H) = 1.2*U*_eq_(C) and 1.5*U*_eq_(C15 and O).

### Crystal data

**Table:**

C_14_H_11_N_3_O_3_·CH_4_O	*F*(000) = 632
*M**_r_* = 301.30	*D*_x_ = 1.367 Mg m^−^^3^
Monoclinic, *P*2_1_/*n*	Mo *K*α radiation, λ = 0.71073 Å
Hall symbol: -P 2yn	Cell parameters from 1170 reflections
*a* = 6.9768 (11) Å	θ = 2.4–21.5°
*b* = 12.2103 (13) Å	µ = 0.10 mm^−^^1^
*c* = 17.2650 (19) Å	*T* = 298 K
β = 95.497 (1)°	Block, yellow
*V* = 1464.0 (3) Å^3^	0.43 × 0.19 × 0.15 mm
*Z* = 4	

### Data collection

**Table:**

Siemens SMART CCD area-detector diffractometer	2508 independent reflections
Radiation source: fine-focus sealed tube	1233 reflections with *I* > 2σ(*I*)
graphite	*R*_int_ = 0.076
φ and ω scans	θ_max_ = 25.0°, θ_min_ = 2.1°
Absorption correction: multi-scan (*SADABS*; Sheldrick, 1996)	*h* = −8→8
*T*_min_ = 0.958, *T*_max_ = 0.985	*k* = −12→14
7290 measured reflections	*l* = −19→20

### Refinement

**Table:**

Refinement on *F*^2^	Primary atom site location: structure-invariant direct methods
Least-squares matrix: full	Secondary atom site location: difference Fourier map
*R*[*F*^2^ > 2σ(*F*^2^)] = 0.050	Hydrogen site location: inferred from neighbouring sites
*wR*(*F*^2^) = 0.141	H-atom parameters constrained
*S* = 0.99	*w* = 1/[σ^2^(*F*_o_^2^) + (0.0559*P*)^2^] where *P* = (*F*_o_^2^ + 2*F*_c_^2^)/3
2508 reflections	(Δ/σ)_max_ < 0.001
199 parameters	Δρ_max_ = 0.34 e Å^−^^3^
0 restraints	Δρ_min_ = −0.19 e Å^−^^3^

### Fractional atomic coordinates and isotropic or equivalent isotropic displacement parameters (Å^2^)

**Table:**

	*x*	*y*	*z*	*U*_iso_*/*U*_eq_	
N1	0.2762 (4)	0.52906 (18)	0.52873 (12)	0.0371 (7)	
H1	0.3076	0.5854	0.5570	0.045*	
N2	0.2993 (4)	0.42528 (18)	0.55868 (14)	0.0390 (7)	
N3	0.1111 (4)	0.8638 (2)	0.36585 (14)	0.0438 (7)	
O1	0.1643 (3)	0.46359 (16)	0.41058 (12)	0.0537 (7)	
O2	0.5588 (3)	0.46550 (16)	0.77984 (11)	0.0523 (7)	
H2	0.5752	0.5125	0.8137	0.078*	
O3	0.4040 (4)	0.38081 (18)	0.86917 (13)	0.0654 (8)	
O4	0.4701 (4)	0.3132 (2)	0.38940 (15)	0.0876 (10)	
H4	0.3697	0.3467	0.3775	0.131*	
C1	0.2034 (5)	0.5407 (2)	0.45415 (17)	0.0361 (8)	
C2	0.1221 (5)	0.8462 (3)	0.44169 (18)	0.0474 (9)	
H2A	0.1103	0.9057	0.4745	0.057*	
C3	0.1501 (5)	0.7440 (2)	0.47459 (17)	0.0406 (9)	
H3	0.1551	0.7347	0.5282	0.049*	
C4	0.1704 (4)	0.6558 (2)	0.42630 (15)	0.0312 (7)	
C5	0.1561 (5)	0.6736 (2)	0.34726 (16)	0.0380 (8)	
H5	0.1660	0.6155	0.3131	0.046*	
C6	0.1271 (5)	0.7785 (3)	0.31942 (18)	0.0429 (9)	
H6	0.1183	0.7900	0.2659	0.052*	
C7	0.3532 (4)	0.4180 (2)	0.63048 (16)	0.0347 (8)	
H7	0.3768	0.4809	0.6602	0.042*	
C8	0.3783 (4)	0.3093 (2)	0.66650 (17)	0.0322 (8)	
C9	0.4202 (5)	0.2943 (2)	0.74724 (17)	0.0353 (8)	
C10	0.4338 (5)	0.1888 (2)	0.77718 (19)	0.0456 (9)	
H10	0.4572	0.1786	0.8306	0.055*	
C11	0.4133 (5)	0.0993 (3)	0.7289 (2)	0.0548 (10)	
H11	0.4255	0.0290	0.7496	0.066*	
C12	0.3750 (5)	0.1135 (3)	0.6503 (2)	0.0551 (10)	
H12	0.3624	0.0530	0.6175	0.066*	
C13	0.3551 (5)	0.2177 (2)	0.61980 (18)	0.0434 (9)	
H13	0.3254	0.2264	0.5665	0.052*	
C14	0.4585 (5)	0.3846 (3)	0.80510 (18)	0.0416 (9)	
C15	0.4316 (6)	0.2034 (3)	0.3937 (2)	0.0742 (13)	
H15A	0.4264	0.1828	0.4471	0.111*	
H15B	0.5314	0.1626	0.3720	0.111*	
H15C	0.3101	0.1879	0.3648	0.111*	

### Atomic displacement parameters (Å^2^)

**Table:**

	*U*^11^	*U*^22^	*U*^33^	*U*^12^	*U*^13^	*U*^23^
N1	0.0488 (19)	0.0284 (14)	0.0325 (15)	0.0004 (13)	−0.0045 (13)	0.0009 (11)
N2	0.0479 (19)	0.0308 (14)	0.0365 (15)	−0.0022 (13)	−0.0049 (13)	0.0049 (12)
N3	0.046 (2)	0.0452 (16)	0.0389 (16)	0.0059 (14)	−0.0003 (13)	0.0045 (13)
O1	0.076 (2)	0.0369 (12)	0.0436 (13)	0.0013 (12)	−0.0172 (12)	−0.0072 (11)
O2	0.078 (2)	0.0419 (13)	0.0375 (13)	−0.0119 (13)	0.0066 (12)	−0.0091 (10)
O3	0.099 (2)	0.0622 (16)	0.0371 (14)	0.0029 (15)	0.0188 (14)	0.0043 (12)
O4	0.091 (3)	0.0691 (19)	0.096 (2)	−0.0088 (17)	−0.0259 (17)	−0.0136 (15)
C1	0.041 (2)	0.0362 (18)	0.0302 (17)	0.0006 (16)	−0.0025 (15)	−0.0011 (14)
C2	0.063 (3)	0.045 (2)	0.0339 (19)	0.0114 (18)	0.0027 (16)	−0.0026 (15)
C3	0.055 (3)	0.0425 (19)	0.0232 (17)	0.0076 (17)	0.0002 (16)	0.0029 (14)
C4	0.030 (2)	0.0366 (17)	0.0266 (16)	0.0033 (14)	0.0013 (13)	−0.0005 (13)
C5	0.041 (2)	0.0438 (19)	0.0284 (17)	0.0048 (16)	0.0018 (15)	−0.0019 (14)
C6	0.046 (2)	0.054 (2)	0.0286 (18)	0.0026 (18)	−0.0005 (16)	0.0094 (16)
C7	0.041 (2)	0.0315 (17)	0.0309 (17)	−0.0005 (15)	−0.0007 (14)	0.0031 (13)
C8	0.028 (2)	0.0290 (17)	0.0388 (18)	−0.0009 (14)	0.0007 (14)	0.0024 (14)
C9	0.034 (2)	0.0332 (17)	0.0374 (18)	0.0021 (15)	−0.0006 (15)	0.0079 (14)
C10	0.051 (2)	0.041 (2)	0.044 (2)	0.0005 (17)	0.0033 (17)	0.0115 (16)
C11	0.061 (3)	0.0333 (19)	0.069 (3)	−0.0039 (18)	0.001 (2)	0.0131 (18)
C12	0.067 (3)	0.032 (2)	0.066 (2)	−0.0038 (18)	0.001 (2)	−0.0039 (18)
C13	0.048 (3)	0.0372 (19)	0.0430 (19)	−0.0029 (17)	−0.0059 (17)	−0.0002 (15)
C14	0.050 (2)	0.0410 (19)	0.0329 (18)	0.0089 (18)	0.0001 (16)	0.0054 (15)
C15	0.085 (4)	0.058 (3)	0.077 (3)	−0.005 (2)	−0.004 (2)	−0.007 (2)

### Geometric parameters (Å, °)

**Table:**

N1—C1	1.346 (3)	C5—C6	1.376 (4)
N1—N2	1.372 (3)	C5—H5	0.9300
N1—H1	0.8600	C6—H6	0.9300
N2—C7	1.264 (3)	C7—C8	1.469 (4)
N3—C2	1.322 (4)	C7—H7	0.9300
N3—C6	1.325 (4)	C8—C13	1.380 (4)
O1—C1	1.219 (3)	C8—C9	1.409 (4)
O2—C14	1.309 (4)	C9—C10	1.388 (4)
O2—H2	0.8200	C9—C14	1.495 (4)
O3—C14	1.205 (3)	C10—C11	1.374 (4)
O4—C15	1.371 (4)	C10—H10	0.9300
O4—H4	0.8200	C11—C12	1.369 (4)
C1—O1	1.219 (3)	C11—H11	0.9300
C1—C4	1.496 (4)	C12—C13	1.378 (4)
C2—C3	1.377 (4)	C12—H12	0.9300
C2—H2A	0.9300	C13—H13	0.9300
C3—C4	1.377 (4)	C15—H15A	0.9600
C3—H3	0.9300	C15—H15B	0.9600
C4—C5	1.376 (4)	C15—H15C	0.9600
			
C1—N1—N2	118.6 (2)	C8—C7—H7	120.3
C1—N1—H1	120.7	C13—C8—C9	118.3 (3)
N2—N1—H1	120.7	C13—C8—C7	118.9 (3)
C7—N2—N1	116.6 (2)	C9—C8—C7	122.8 (3)
C2—N3—C6	118.1 (3)	C10—C9—C8	119.3 (3)
C14—O2—H2	109.5	C10—C9—C14	115.7 (3)
C15—O4—H4	109.5	C8—C9—C14	124.9 (3)
O1—C1—N1	123.4 (3)	C11—C10—C9	120.9 (3)
O1—C1—N1	123.4 (3)	C11—C10—H10	119.6
O1—C1—C4	120.6 (3)	C9—C10—H10	119.6
O1—C1—C4	120.6 (3)	C12—C11—C10	120.0 (3)
N1—C1—C4	116.0 (3)	C12—C11—H11	120.0
N3—C2—C3	123.3 (3)	C10—C11—H11	120.0
N3—C2—H2A	118.4	C11—C12—C13	119.9 (3)
C3—C2—H2A	118.4	C11—C12—H12	120.0
C2—C3—C4	118.5 (3)	C13—C12—H12	120.0
C2—C3—H3	120.7	C12—C13—C8	121.6 (3)
C4—C3—H3	120.7	C12—C13—H13	119.2
C5—C4—C3	118.3 (3)	C8—C13—H13	119.2
C5—C4—C1	117.5 (2)	O3—C14—O2	124.1 (3)
C3—C4—C1	124.2 (2)	O3—C14—C9	122.2 (3)
C6—C5—C4	119.2 (3)	O2—C14—C9	113.7 (3)
C6—C5—H5	120.4	O4—C15—H15A	109.5
C4—C5—H5	120.4	O4—C15—H15B	109.5
N3—C6—C5	122.5 (3)	H15A—C15—H15B	109.5
N3—C6—H6	118.7	O4—C15—H15C	109.5
C5—C6—H6	118.7	H15A—C15—H15C	109.5
N2—C7—C8	119.4 (3)	H15B—C15—H15C	109.5
N2—C7—H7	120.3		

### Hydrogen-bond geometry (Å, °)

**Table:**

*D*—H···*A*	*D*—H	H···*A*	*D*···*A*	*D*—H···*A*
N1—H1···O4^i^	0.86	2.13	2.891 (3)	148
O4—H4···O1	0.82	2.14	2.864 (4)	148
O2—H2···N3^ii^	0.82	1.76	2.565 (3)	165

Symmetry codes: (i) −*x*+1, −*y*+1, −*z*+1;  (ii) *x*+1/2, −*y*+3/2, *z*+1/2.
